# Different Trafficking Phenotypes of Niemann-Pick C1 Gene Mutations Correlate with Various Alterations in Lipid Storage, Membrane Composition and Miglustat Amenability

**DOI:** 10.3390/ijms21062101

**Published:** 2020-03-19

**Authors:** Graham Brogden, Hadeel Shammas, Friederike Walters, Katia Maalouf, Anibh M. Das, Hassan Y. Naim, Sandra Rizk

**Affiliations:** 1 Department of Physiological Chemistry, University of Veterinary Medicine Hannover, 30559 Hannover, Germany; graham.brogden@twincore.de (G.B.); shammas.hadeel@mh-hannover.de (H.S.); friederike.walters@tiho-hannover.de (F.W.); katia.maalouf@lau.edu.lb (K.M.); hassan.naim@tiho-hannover.de (H.Y.N.); 2 Department of Paediatrics, Hannover Medical School, Carl-Neuberg-Strasse 1, 30625 Hannover, Germany; das.anibh@mh-hannover.de; 3 Department of Natural Sciences, Lebanese American University, Beirut 1102-2801, Lebanon

**Keywords:** Niemann-Pick type C, cholesterol, Miglustat (N-butyldeoxynojirimycin; NB-DNJ), lipid rafts

## Abstract

Niemann-Pick Type C (NPC) is an autosomal recessive lysosomal storage disease leading to progressive neurodegeneration. Mutations in the *NPC1* gene, which accounts for 95% of the cases, lead to a defect in intra-lysosomal trafficking of cholesterol and an accumulation of storage material including cholesterol and sphingolipids in the endo-lysosomal system. Symptoms are progressive neurological and visceral deterioration, with variable onset and severity of the disease. This study investigates the influence of two different NPC1 mutations on the biochemical phenotype in fibroblasts isolated from NPC patients in comparison to healthy, wild type (WT) cells. Skin derived fibroblasts were cultured from one patient compound-heterozygous for D874V/D948Y mutations, which presented wild-type like intracellular trafficking of NPC1, and a second patient compound- heterozygous for I1061T/P887L mutations, which exhibited a more severe biochemical phenotype as revealed in the delayed trafficking of NPC1. Biochemical analysis using HPLC and TLC indicated that lipid accumulations were mutation-dependent and correlated with the trafficking pattern of NPC1: higher levels of cholesterol and glycolipids were associated with the mutations that exhibited delayed intracellular trafficking, as compared to their WT-like trafficked or wild type (WT) counterparts. Furthermore, variations in membrane structure was confirmed in these cell lines based on alteration in lipid rafts composition as revealed by the shift in flotillin-2 (FLOT2) distribution, a typical lipid rafts marker, which again showed marked alterations only in the NPC1 mutant showing major trafficking delay. Finally, treatment with N-butyldeoxynojirimycin (NB-DNJ, Miglustat) led to a reduction of stored lipids in cells from both patients to various extents, however, no normalisation in lipid raft structure was achieved. The data presented in this study help in understanding the varying biochemical phenotypes observed in patients harbouring different mutations, which explain why the effectiveness of NB-DNJ treatment is patient specific.

## 1. Introduction

Niemann-Pick Type C disease (NPC, OMIM #257220) is a progressive neurodegenerative lysosomal disorder that is inherited in a rare autosomal recessive pattern from mutations in *NPC1* or *NPC2* genes. NPC disease has an incidence of approximately 1:90,000, with 95% due to a mutation in the *NPC1* gene and the remaining 5% in the *NPC2* gene. A late onset form of the disease with a milder clinical phenotype has also been estimated to have an occurrence of up to 1:19,000 [1]. NPC disease is a progressive neurodegenerative disorder with over 400 reported mutations, resulting in a wide diversity of clinical manifestations. The most common mutation is isoleucine to threonine in position 1061 (I1061T) that affects almost 20% of Western European patients [2].

NPC1 is a multispan membrane glycoprotein that comprises 1273 amino acids, constituting the two luminal domains, the cholesterol binding domain and a cysteine-rich loop, a cytosolic sterol-sensing domain and 13 transmembrane domains [3]. NPC1 is synthesised and co-translationally N-glycosylated in the ER, trafficked after acquisition of correct folding to the Golgi apparatus, where it matures to a complex glycosylated protein that is ultimately transported via the endosomal/lysosomal system to the lysosomes [4]. Here, a complex of NPC1-cholesterol-NPC2 is formed facilitating the transport of cholesterol out of the endosomal/lysosomal compartment [5].

NPC is characterised by accumulations of specific lipids, primarily cholesterol and glycosphingolipids, in a broad range of cell types. A mutation in either NPC protein leads to a defect in the trafficking of unesterified cholesterol out of the lysosomes, leading to lipid accumulations and subsequent cellular malfunction [2]. Lipid accumulation has been shown to be tissue specific, with cholesterol, sphingomyelin and sphingosine accumulation being most abundant in peripheral tissues, while glycosphingolipid storage is most prominent in the central nervous system [6]. In an in vitro setup, similar lipid accumulations have also been described. Patient skin-derived fibroblasts are routinely used to diagnose NPC disease by staining unesterified cholesterol with filipin, which reveal varying levels of staining between classical and variant forms of NPC disease compatible with mutation-specific lipid accumulations [7]. Nevertheless, filipin staining per se is not a sufficient diagnostic approach and requires confirmation by genetic testing [8]. Accumulation of cholesterol is not the only feature of NPC. In fact, many other lipids are also increased, most notably (glyco-) sphingolipids, diacylglycerol, phospholipids, sterols and ceramides [9,10]. Until present, detailed investigations that correlate specific mutations with the protein and ultimately clinical phenotypes have been scarce. Recently, a comprehensive study with a panel of NPC1 mutations has established the concept of phenotypic heterogeneity of the NPC1 mutants at the biochemical and cellular levels, and correlated the trafficking classes of the NPC1 mutants with their clinical phenotypes and the severity of symptoms [11]. Nevertheless, these studies have been performed in a heterologous cellular model with individual NPC1 mutants and not as they occur in vivo as homozygous, compound-heterozygous or even heterozygous.

The aim of this study was to determine the mutation-dependent biochemical phenotypes in fibroblasts from two NPC patients harbouring different compound-heterozygous NPC 1 mutations, to further correlate the trafficking phenotype to membrane composition and levels of lipid storage. Additionally, the influence of the iminosugar NB-DNJ (Miglustat) on the accumulation of lipids and membrane alterations in the patients and control cell lines was studied in vitro. The data show a correlation between the trafficking pattern of NPC1, levels of lipid accumulation, membrane composition and the response to NB-DNJ incubation.

## 2. Results

### 2.1. Variations in the Trafficking Behaviour of NPC1 Mutants

**Figure 1:**
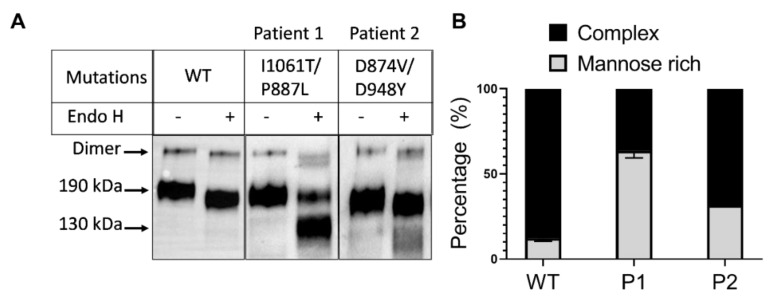
Homogenates were immunoprecipitated with an anti-NPC1 antibody, and the resulting sample was divided into two, one untreated (-) and the second treated with EndoH (+). The complex glycosylated EndoH-resistant form of the NPC protein can be seen at 190 kDa and the EndoH-sensitive form at 130 kDa (**A**). Bands corresponding to the complex glycosylated and mannose rich forms of the NPC protein were quantified and are displayed as a percentage of the total (**B**).

The trafficking behaviour of NPC1 in fibroblasts taken from NPC patients was examined by testing the sensitivity of NPC1 protein to Endo H. Endo H distinguishes between the mannose-rich immature form of proteins that are located in the ER or in the early secretory pathway and their complex glycosylated mature counterparts that have been processed in the Golgi apparatus. As previously shown in transfected COS-1 cells [11] wild type NPC1 appeared as a diffuse band at about 190 kDa that was only slightly sensitive to Endo H corresponding therefore to complex glycosylated NPC1 (Figure 1, left two lanes). NPC1 expressed in the patients’ fibroblasts exhibited different patterns upon Endo treatment. Patient 1 is compound-heterozygous carrying I1061T/P887L mutations, with I1061T being the most common mutation in NP-C. When the NPC1 mutant carrying the I1061T mutation was expressed individually in COS-1 cells, a mannose-rich Endo H-sensitive protein was detected that was completely blocked in the ER [11]. Similarly, the P887L also revealed the same ER block trafficking phenotype (Supplementary Figure S1). We therefore wanted to identify the biosynthetic forms of NPC1 in fibroblasts expressing the two mutations. As shown in Figure 1, NPC1 in patient 1 fibroblasts comprises two glycoforms of varying staining intensities: an approximately 130 kDa protein that is derived from the Endo H-sensitive mannose-rich ER located NPC1, and another less heavily stained 190 kDa endo H-resistant mature NPC1 (Figure 1). Furthermore, a specific protein band of a higher size can also be detected that follows a similar Endo H treatment profile, with Endo H-sensitive and -resistant bands proposing this band to represent a dimeric form of NPC1. The different protein trafficking pattern of NPC1 carrying the I1061T/P887L mutations as compared to the same mutants expressed separately in COS-1 cells suggests that a potential interaction between two NPC1 monomers under normal expression levels has taken place in contrast to the overexpression cellular model.

Fibroblasts from patient 2, whose pattern of inheritance is compound-heterozygous for the mutations D874V/D948Y, contained almost exclusively the complex glycosylated NPC1 species and to a substantially lesser extent the Endo H-product of the mannose form of NPC1 (Figure 1). Noteworthy, the two mutations in patient 2 generate a wild type-like trafficking pattern (D948Y) or a partially-trafficked NPC1 (D874V) when individually expressed in COS-1 cells [11]. In view of the overall wild-type NPC1 features of the resultant protein in this patient, a hierarchy can be proposed, in which the wild type-like protein phenotype prevails over the partially trafficked phenotype.

### 2.2. Variable Levels of Lipid Accumulations Correlate with the NPC Mutation and the Trafficking Pattern

**Figure 2:**
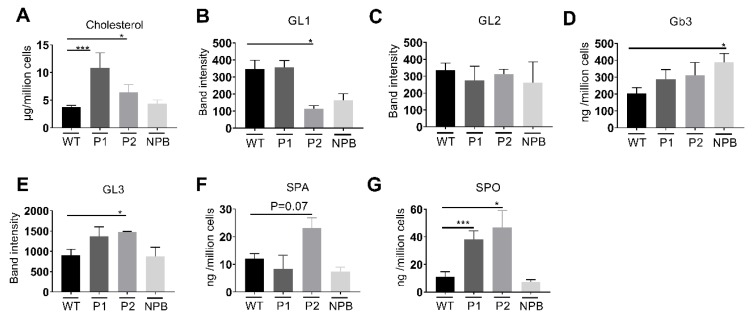
Lipid analysis from total cell lysates. Cholesterol (**A**), sphinganine (**F**: SPA) and sphingosine (**G**: SPO) analysis was performed by HPLC and glycolipid analysis by TLC. Cholesterol and sphingolipid concentrations are given as ng cholesterol per 1 × 10^6^ cells. Four glycolipids were present in significant concentrations, Globotriaosylceramide (**D**: Gb3), glycolipid 1, 2, 3 (**B**: GL1, **C**: GL2 and **E**: GL3). Each bar equates to at least three repetitions, with the wild type (WT) corresponding to the average of three repetitions of three WT fibroblasts from independent healthy donors. P1: NPC-I1061T/P887L, P2: NPC-D874V/D948Y. SEM, Student’s *t*-test * *p* ≤ 0.05, *** *p* ≤ 0.001. *n* = 3–11.

**Figure 3:**
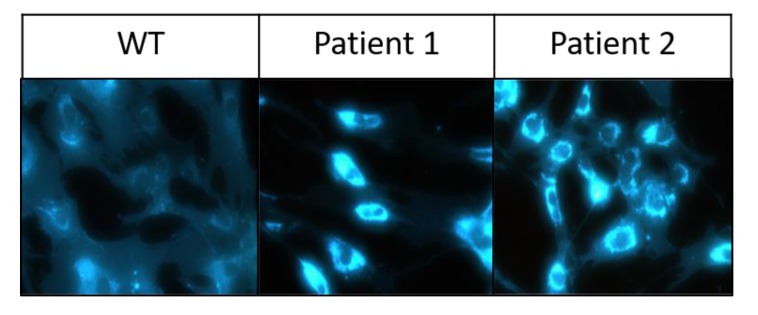
Filipin staining revealed lysosomal cholesterol accumulation in fibroblasts isolated from both NPC patients as compared to the healthy donor (WT). Patient 1: NPC-I1061T/P887L, Patient 2: NPC-D874V/D948Y.

**Figure 4:**
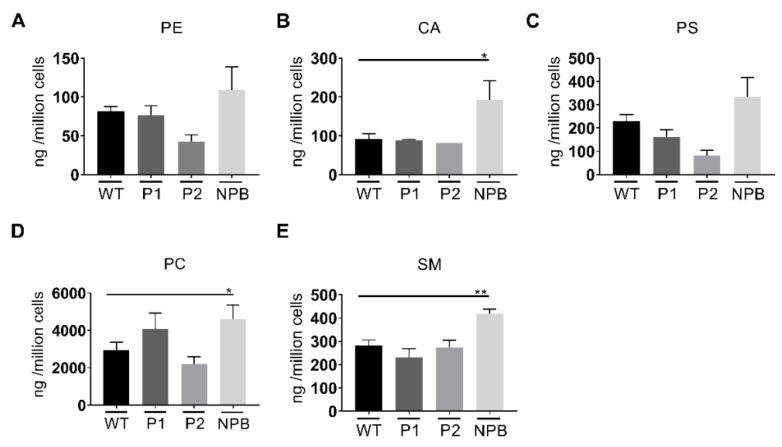
Lipid analysis from total cell lysates. PE (phosphoethanolamine, **A**), CA (cardiolipin, **B**), PC (phosphatidylserine, **C**), PC (phosphatidylcholine, **D**) and SM (sphingomyelin, **E**) were analysed by TLC. Concentrations are given as ng cholesterol per 1 × 10^6^ cells. Each bar equates to at least three repetitions, with the WT corresponding to the average of three repetitions of three WT fibroblasts from independent healthy donors. P1: NPC-I1061T/P887L, P2: NPC-D874V/D948Y. SEM, Student’s *t*-test * *p* ≤ 0.05, ** *p* ≤ 0.01. *n* = 3–11.

Fibroblasts were grown to confluence, lysed prior to lipid extraction and subsequent lipid analysis. The contents of cholesterol and sphingolipids were assessed by HPTLC, while TLC was utilised to measure the levels of glycolipids and phospholipids. Lipid analysis of fibroblasts derived from NPC and Niemann-Pick Type B (NPB) patients showed accumulations in specific lipid groups. The fibroblasts from all three patients studied here showed significantly higher levels of cholesterol in NPC compared to the WT fibroblasts (Figure 2A). Interestingly, in fibroblasts from patient 1, in which the trafficking pattern of NPC1 revealed severe delays, significantly higher cholesterol levels were detected when compared to fibroblasts from patient 2. Cholesterol accumulation in the NPC fibroblasts was also confirmed using filipin staining (Figure 3), whereby the staining in both cell lines was almost the same confirming the view that filipin staining in not quantitative enough or sufficient per se for NPC diagnostics. Fibroblasts isolated from a patient diagnosed with NPB did not show any increase in cholesterol, however, importantly they showed a significant accumulation of sphingomyelin as expected (Figure 4).

Glycolipid (GL) analysis by TLC revealed four GLs in significant concentrations (Figure 2, GL1-GL4, B-E, respectively). Significantly lower levels of an unknown GL1 were shown in patient 2 containing the D874V/D948Y mutations (Figure 2B). The levels of a second glycolipid, GL2 (Figure 2C), which migrated almost as far up the TLC plate as GL1, were not influenced by NPC1 mutations, however increased levels of Gb3 and GL3 (Figure 2D,E), which migrated almost as far as Gb3 on the TLC plate were detected in patient 1, but not patient 2. Sphingosine (SPO) concentrations were shown to be significantly higher in fibroblasts isolated from NPC patients (Figure 2G), whereas elevated levels were evident in the concentration of sphinganine (SPA) (Figure 2F). Lastly, no significant differences in phospholipids were detected between the fibroblasts from NPC patients compared to WT cells (Figure 4).

### 2.3. Membrane Alterations Correlate with the NPC Mutation and the Trafficking Pattern

**Figure 5:**
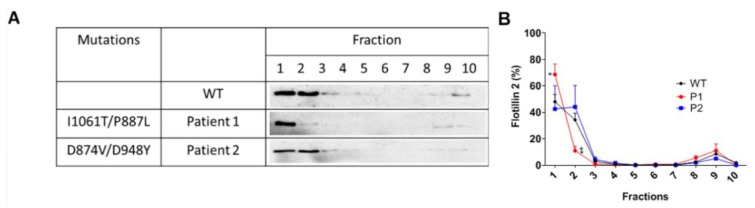
Cells were lysed in Lubrol and separated on a sucrose density gradient. Immunoblotting was subsequently performed to determine the distribution of flotillin-2. Representative immunoblots (**A**) and graphs based on quantification of three independent repetitions (**B**). WT represents at least three repetitions from three WT fibroblasts. SEM, Student’s *t*-test * *p* ≤ 0.05.

In view of the variations in the contents of the membrane lipids, we asked whether cholesterol- and sphinoglipids-enriched membrane microdomains or lipid rafts (LR), which are implicated in several intracellular mechanisms, such as signalling, protein trafficking and sorting, could be also affected in the patients’ fibroblasts. For this purpose, sucrose density gradient centrifugations of cellular detergent extracts were performed. Under normal conditions LR are predominately located in the floating fractions of the gradients, notably in fractions 1–3 that can be assessed by the distribution of FLOT2, a LR-associated signalling protein. FLOT2 was distributed evenly between the top floating fractions 1 and 2 of the gradient, with a reduced appearance in the non LR fractions (Figure 5A,B). On the other hand, fibroblasts from patient 1 (I1061T/P887L) revealed FLOT2 almost exclusively in the first fraction and to a lesser extent in the bottom fractions. Interestingly, patient 2, who exhibited a trafficking pattern of NPC1 similar to the WT form, revealed a FLOT2 distribution pattern similar to that present in the control cells. The variations in the distribution of FLOT2 are concomitant with the altered levels of cholesterol and sphingolipids in the patients’ fibroblasts.

### 2.4. NB-DNJ Reduces Lipid Accumulations in a Concentration- and NPC1 Mutation-Dependent Manner but does not Restore Normal Membrane Structure

**Figure 6:**
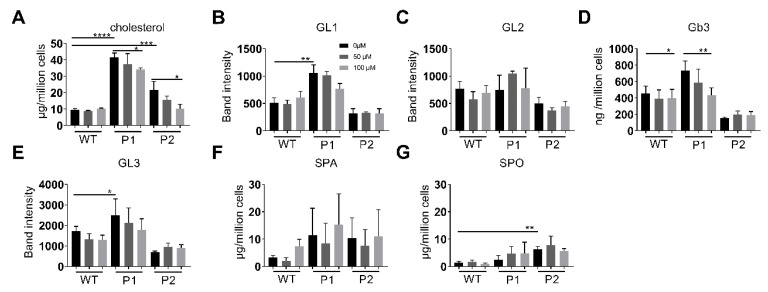
Lipid analysis from total cell lysates upon treatment with 50 and 100 μM NB-DNJ. Cholesterol and glycolipid analysis was performed by HPLC and TLC, respectively. Cholesterol (**A**), GL (Glycolipid, **B**, **C** and **E**), Gb3 (Globotriaosylceramide, **D**), SPA (Sphinganine, **F**), SPO (Sphingosine, **G**). Each bar equates to at least three repetitions, with the WT corresponding to the average of three repetitions of three WT fibroblasts from independent healthy donors. P1: I1061T/P887L, P2: D874V/D948Y. SEM, Student’s *t*-test * *p* ≤ 0.05, ** *p* ≤ 0.01, *** *p* ≤ 0.001, **** *p* ≤ 0.00001. *n* = 3–11.

Substrate reduction therapy using Miglustat is one therapeutic measure in treatment of NP-C. Miglustat or N-butyldeoxynojirimycin (NB-DNJ) is an iminosugar that inhibits the synthesis of sphingolipids, which also accumulate in NP-C, most likely as a secondary effect to cholesterol imbalance in the membranes. We, therefore, aimed in this study to elucidate the effects of NB-DNJ on the levels of cholesterol and sphingolipids in fibroblasts of the two patients versus the wild type control. NB-DNJ treatment of fibroblasts led to a concentration dependant decrease in cholesterol concentrations across fibroblasts isolated from both NPC patients; however, no effect was detected in WT cells (Figure 6A). Furthermore, a general concentration-dependent decrease in the level of glycolipids was evident in the fibroblasts from patient 1 containing the I1061T/P887L mutations, while this effect was not similarly high as in the WT and patient 2 fibroblasts (mutations D874V/D948Y) (Figure 6B–E). Phospholipid concentrations remained unchanged under NB-DNJ incubation (Supplementary Figure S2). SPA and SPO remained unaffected post NB-DNJ treatment (Figure 6F,G).

**Figure 7:**
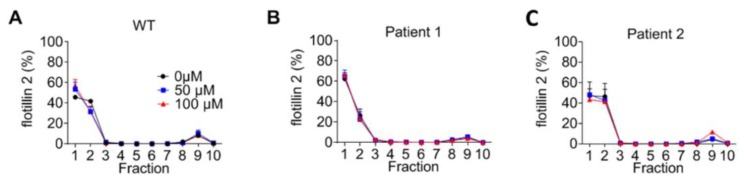
Flotillin-2 distribution in 10 fractions isolated from a sucrose density gradient. Fibroblasts (**A**: WT, **B**: I1061T/P887L, **C**: D874V/D948Y) were grown to confluence and treated with 50 µM, 100 µM or untreated for three days. Cells were lysed in Lubrol and separated on a sucrose density gradient. Immunoblotting was subsequently performed to determine the distribution of flotillin-2. Graphs based on the quantification of at least three independent repetitions. Results are expressed as a percentage of the total amount of flotillin 2. SEM, Student’s *t*-test * *p* ≤ 0.05.

Finally, the effect of NB-DNJ on lipid rafts was investigated. WT fibroblasts treated for three days with NB-DNJ showed a shift in FLOT2 localisation from the second to the lighter first gradient fraction (Figure 7A). However, no change in FLOT2 distribution was evident after three days of NB-DNJ treatment (Figure 7A–C).

## 3. Discussion

NPC disease is a complex multifaceted lysosomal neurodegenerative condition with varyingly poor prognosis. We have recently elucidated structural and biosynthetic features of a panel of NPC1 mutations and classified these according to their trafficking pattern and intracellular localizations into three major phenotypes that vary in their biochemical severity [11]. Despite the large body of knowledge on the genetic background and the clinical features of several NPC1 mutations, no studies at present are known on the interaction of NPC1 mutants with each other, whether as homozygotes or compound-heterozygotes, and the implication of this interaction on the resulting NPC1 phenotype. The trafficking behaviour of NPC1 in the patients’ fibroblasts harbouring the I1061T/P887L mutations showed trafficking phenotypes compatible with intracellular localisation in the ER and partial processing in the Golgi. Almost 30% of the resulting NPC1 in the patients’ fibroblasts were converted into a mature complex glycosylated Endo H-resistant form. This pattern differs from that observed in transfected COS-1 cells that have been individually transfected with cDNA encoding the NPC1 carrying either I1061T or P887L mutations: in both individual cases the mutations have elicited an intracellular trafficking arrest of the NPC1 mutant in the ER [11]. It is likely that, in a fashion similar to wild type NPC1, an interaction between the monomeric forms of the NPC1 mutants takes place in the patients’ fibroblasts generating a dimeric protein species that exhibits similar endo H-sensitivity pattern to the monomeric 190-kDa form. This dimeric form is apparently highly stable, since it can be still detected in both wild type NPC1 and the NPC1 forms from the patients’ fibroblasts, despite the denaturing conditions utilised in the electrophoretic analysis. It is possible that the mutants assume a thermodynamically favourable conformation that permits their interaction and generates a trafficking competent dimer that exits the ER. Several proteins are packaged in COPII vesicles when they have acquired dimeric or higher order conformation [12]. Even in mutant proteins, a minimal folding may permit the protein to escape the quality control mechanism in the ER [13,14].

The compound-heterozygous D874V/D948Y mutations revealed virtually normal processing in the Golgi. Recently, it has been shown that trafficking of NPC1 mutant carrying the D874V mutation resembles that of wild type NPC1, while NPC1 carrying the mutation D948Y is only partially transported along the secretory pathway. The combination of the two mutants in the fibroblasts from patient 2 generates a protein that reveals trafficking features almost similar to wild type NPC1 protein suggesting a hierarchy of the wild type-like phenotype versus the other mutant.

Recent work has shown that cells devoid of NPC1 accumulate a wide range of lipid groups [9]; however, the correlation between different mutations and membrane lipid composition still needs to be resolved. Our study shows that lipid accumulations, specifically cholesterol and glycolipids, are significantly higher in fibroblasts derived from both patients as compared to the control cells. Interestingly, a greater accumulation was found in fibroblasts isolated from patient 1, harbouring the I1061T and P887L mutations, both mutations lead to a severe delay in the trafficking pattern of NPC1; the accumulation is almost similar to that found in NPC1-deficient CHO cells (Figure S3) concomitant with malfunctioning of the mutants NPC1 in these fibroblasts. Likewise, the increased levels of Gb3 and GL3 glycolipids conform to the overall accumulative pattern of lipids in NPC patients and not only cholesterol. Furthermore, Harzer et al. reported an average 2.5-fold increase in Gb3 isolated from NPC patient fibroblasts, however, interestingly not every patient analysed showed elevated Gb3 levels [15].

Lipid modulations have also been shown to lead to membrane, and in particular LR, alterations in lysosomal storage diseases, contributing to the pathophysiology of the diseases [16]. Membrane alterations have previously been described in lysosomal storage diseases, such as Fabry [17] and Gaucher [18]. Our data support the notion that LR alterations, as assessed by FLOT2 distribution, correlate well with the trafficking pattern of NPC1. Several cellular functions, such as protein trafficking, sorting and endocytosis, are associated with LR rendering an alteration in the LR composition crucial for the cellular homeostasis. Previous studies with intestinal biopsy specimens from an NPC patient expressing the I1061T mutation have reported an impaired intracellular trafficking and functional deficits of the LR associated proteins sucrase-isomaltase and maltase-glucoamylase, explaining thus the reduced carbohydrate digestion in the intestinal lumen and delineating the effect of deficient cholesterol and sphingolipid homeostasis in development of gastrointestinal symptoms in NPC patients [19]. While these enzymes are directly trafficked to the apical membrane in intestinal cells, another enzyme, dipeptidyl peptidase IV (DPPIV), a lipid raft associated protein [20] is trafficked along the transcytosis pathway [21] and undergoes endocytosis. Here also, DPPIV is endocytosed in wild type fibroblasts, but this event is substantially hampered in fibroblasts from patient 1 as shown by the heavy accumulation of the protein at the cell surface (Supplementary Figures S4 and S5). These results add further support to the systemic delay of intracellular processing and trafficking of proteins in NPC cells due to distorted LR structure and, consequently, function [22].

The data presented in this communication show a clear correlation between the severity in trafficking impairment, lipid accumulation and membrane alterations. However, how this translates to the effectiveness of therapeutic treatments remains unclear. It is well established that NPC patients respond differently to treatment with NB-DNJ [19]. NB-DNJ has been shown to act as chaperone for beta-glucosidase in Gaucher disease, where it potentially increases enzyme activity and reduces degradation by stabilising the structure of the enzyme [23]. A similar chaperone function could also be present for NB-DNJ in NPC disease, however, further investigations need to be performed to determine if proteasome associated degradation of NPC1 in the cells is inhibited. Recent research by Schultz et al. showed that the degradation half-life of wild type NPC1 in the ER is approximately 9 h, whilst the NPC1 mutant carrying the I1061T is only 6.5 h [22]. NB-DNJ has also been shown to have chaperone capabilities [23], thus, it can be hypothesised that NB-DNJ treatment may be able to increase the amount of properly trafficked NPC1. This could be especially the case for the NPC1 mutant carrying the D874V and D948Y mutations, where trafficking of NPC1 and cholesterol accumulation are not as severe as compared to the I1061T/P887L mutations. This hypothesis was supported by the greater percentage decrease in cholesterol in the fibroblasts from patient 2 (D874V/D948Y mutations) as compared to those harbouring the I1061T/P887L mutations. However further experiments will need to be conducted both in vitro and in vivo to prove this hypothesis.

NB-DNJ treatment of NPC fibroblasts for three days resulted in a significant decrease in lipid concentrations, primarily cholesterol, and glycolipids to a lesser extent, however, the relative concentrations compared to WT cells remained high. This is in line with the efficacy of NB-DNJ in slowing down the progression of NPC rather than reversing the symptoms. Moreover, the fibroblasts from the patient presenting the more severe trafficking alterations responded less strongly to treatment when compared to patient 2. Interestingly, sphingosine levels were not significantly reduced by NB-DNJ treatment, whereby the inability of NB-DNJ to significantly reduce sphingosine may hinder cholesterol expulsion from lysosomes [24]. Further experiments should be conducted to determine if additional sphingosine depletion can aid the NB-DNJ induced cholesterol reduction in patients harbouring the D874V/D948Y mutations. Even though short-term NB-DNJ treatment was able to reduce some lipid accumulation, it was not able to restore membrane LR abnormalities, namely FLOT2 distribution. LR normalisation by NB-DNJ was previously demonstrated in Triton X-100-resistant LR, which efficiently solubilises the inner membrane, whereas in this study, Lubrol resistant LR were analysed, resulting in a relatively lower cholesterol content since Lubrol is unable to solubilise the inner leaflet of lipid rafts [25]. It is noteworthy that the limited efficacy in LR normalisation reported here is not conclusive, since longer treatment times, both in vitro and in vivo, need to be investigated.

In summary, correlations between the genetic and biochemical phenotype were investigated based on three criteria: lipid accumulations, membrane LR alterations and intracellular trafficking of NPC1. Furthermore, NB-DNJ was efficient at reducing lipid accumulations, without normalising plasma membrane alterations, particularly LRs. The work presented here details the effect of NB-DNJ treatment on two NPC1 mutations and may offer insights into patient-tailored treatment.

## 4. Materials and Methods

### 4.1. Cell Culture

Skin biopsies from NPC and NPB patients (see Table 1) were primarily taken for diagnostic purposes. Patients gave informed consent for using the cells for scientific purposes. The ethical review board of Hannover Medical School has granted a positive vote (EC Nr. 5176) for studying metabolism in human fibroblasts. NPB fibroblasts are a non-cholesterol storing, lysosomal storage disease used for comparison. Control fibroblasts were obtained from age-matched patients undergoing small routine surgery after informed consent. Fibroblasts were grown to confluence in 60 × 15 mm dishes in Dulbecco’s Modified Eagle’s Medium low glucose medium with 10% foetal calf serum and antibiotics at 37 °C in 5% CO_2_. Cells were treated with either 0, 50 or 100 mM NB-DNJ for three days, with fresh medium and NB-DNJ was added every 24 h. Low passage numbers were used throughout the study.

### 4.2. Filipin Staining

Fibroblasts from NPC patients and control individuals were seeded on gelatine-precoated coverslips, incubated for two days at 37 °C and washed thereafter twice with ice-cold PBS. The steps that followed were all performed at room temperature. Here, the cells were fixed with 3% paraformaldehyde (PFA) for 20 min, followed by quenching with 50 mM NH_4_Cl for 30 min and finally permeabilised with 0.2% TritonX-100 for further 30 min. Treatment with the cholesterol-binding reagent Filipin (Sigma Aldrich, subsidiary of Merck KGaA St. Louis, Missouri USA) was performed for 2 h in the dark. The images were visualised using a fluorescence microscope (Leica DM IRB).

### 4.3. Cell Lysis, Immunoprecipitation, Deglycosylation and Immunoblotting

Cell lysates were prepared and immunoprecipitated as described previously with minor alterations [27]. Briefly, NB-DNJ treated and untreated fibroblasts were lysed and the cell lysates were immunoprecipitated with anti-NPC1 antibodies (1:1200, Novus Biologicals, Littleton, CO, USA). The immunoprecipitates were treated or non-treated with endoglycosidase H (Endo H), analysed by SDS-PAGE on 12% slab gels and prepared for Western blotting using PVDF membranes as described previously [28]. Secondary anti-rabbit or anti-mouse antibody conjugated to horseradish peroxidase were used (0.2 μg/mL, ThermoScientific, Schwerte, Germany). Protein bands were visualised using SuperSignal™ West Femto maximum sensitivity Western blot chemiluminescence substrate (Schwerte, Germany) and a ChemiDoc XRS System (Bio-Rad) as described previously [17].

### 4.4. Lipid Analysis

Two million fibroblasts were washed and lysed in PBS by freeze thawing and passing through a 26 G needle 20 times on ice. Lipids were isolated from gradient fractions and cell lysates for cholesterol, phospholipid and glycolipid analysis by a previously published method [29]. Briefly, 2 mL of methanol and 1 mL of chloroform were added to the sample and left to rotate at room temperature for 30 min. Samples were then centrifuged at 600× *g* for 5 min at 7 °C and the subsequent protein pellet discarded. A total of 1 mL of chloroform and 1 mL of distilled water was added to the supernatant, mixed briefly, and centrifuged again as previously stated. The upper phase was removed and the lower phase dried via vacuum drier. The samples were then redissolved in 250 µL of chloroform and methanol solution (1:1) Cholesterol was quantified by HPLC as previously described [17], using a Hitachi Chromaster HPLC system fitted with a Chromolith^®^ HighResolution RP-18 endcapped 100-4.6 mm column coupled to a 5-4.6 mm guard cartridge. The following conditions were used; methanol mobile phase at a flow rate of 1 mL/min at 22 bar and the UV detector set at 202 nm. Quantification was performed against an external standard. Phospholipids and glycolipids were quantified by HPTLC and TLC using previously published methods in [29,30], respectively. Briefly, phospholipids were separated on Silica gel 60 plates (Sigma Aldrich, Missouri USA) using three running solvents, stained using 7.5% phosphoric acid and subsequently baked at 170 °C for 10 min. Glycolipids were separated on Silica gel 60F_254_ plates (Sigma Aldrich, Missouri USA) using a solution of chloroform, methanol and distilled water (65:35:8). The plates were then stained in a solution consisting of 5% orcinol monohydrate (Sigma Aldrich, Missouri USA), 10% sulphuric acid and 85% distilled water, and subsequently baked at 115 °C for 15 min. The TLC plates were then scanned and analysed using CP ATLAS software (Lazarsoftware). Sphinganine and sphingosine were quantified using HPLC coupled to a fluorescence detector as described previously [31]. The samples were quantified against external sphinganine and sphingosine standards, which were emitted at 4.8 and 6.2 min, respectively. All results are displayed as µg lipid/1 × 10^6^ cells.

### 4.5. Isolation of Lipid Rafts

LR were prepared using a previously described protocol with minor alterations [11]. Briefly, cells grown to confluence in 60 × 15 mm dishes and treated for 72 h with NB-DNJ at concentrations of 50 or 100 µM. All procedures were performed on ice or at 4 °C. Cells (2 × 10^6^) were solubilised in 1% Lubrol in 50 mM Tris, 150 mM NaCl, pH 7.6 and 50 µL protease inhibitors. Homogenisation was performed by passing the cell lysate 20 times through a 21 G needle. The lysates were then centrifuged at 1500× *g* for 20 min at 4 °C and the supernatant loaded onto a discontinuous sucrose gradient as described before. Gradients were centrifuged at 4 °C for 18 h at 100,000× *g* using a Beckman SW40 rotor. Ten fractions were collected on ice from top to bottom, with fractions 1–3 containing predominately the LRs fractions, and 8–10 containing predominately the detergent-soluble cell fraction. The sucrose content was quantified from each gradient fraction by using a HI 96801 refractometer (HANNA instruments). The quality and location of LR was assessed by Western blotting of the gradient fractions using anti-FLOT2 antibody (1:5000; sc-28320 Santa Cruz, 200µg/mL, Santa Cruz, California USA).

## 5. Conclusions

In summary, correlations between the genetic and biochemical phenotype were investigated based on three criteria: lipid accumulations, membrane lipid rafts alterations and intracellular trafficking of NPC1. Furthermore, NB-DNJ was efficient at reducing lipid accumulations, without normalising plasma membrane alterations, particularly LRs. The work presented here details the effect of NB-DNJ treatment on two NPC1 mutations in vitro and offers possible insights into patient-tailored treatment.

## Figures and Tables

**Table 1:** Compilation of the fibroblast cell lines from controls and patients, the corresponding mutations, genotypes and protein trafficking phenotypes.

Cell Line	Mutation cDNA	Mutation Protein	Genotype	Protein Trafficking Phenotype	Reference
2821/10 (Control) 2713 (Control) 568/10 (Control)	N/A			Wild type	
12/13 (Patient 1)	3182T>C/3337C>T/	p. I1061T/ p. P887L	Compound-heterozygous	ER block/ ER block	[26] (Figure S1)
493/09 (Patient 2)	2621A>T/ 2846 G>T	p. D874V/ p. D948Y	Compound-heterozygous	Partial trafficking/ wild type like	[26]
79/16 (NPB)	c.481dupC/ c.1829_1831delGCC	p.Leu161Profs*32/ p.Arg610_His611delinsHis	Compound-heterozygous	ND	

## Supplementary Materials

The following are available online at https://www.mdpi.com/1422-0067/21/6/2101/s1

## Abbreviations

FLOT2	Flotillin 2
Gb3	Globotriaosylceramide
GL	Glycolipid
HPLC	High performance liquid chromatography
LR	Lipid rafts
NPB	Niemann-Pick type B
NPC	Niemann-Pick type C
NB-DNJ	N-Butyl Deoxynojirimycin
PBS	Phosphate buffered saline
TLC	Thin layer chromatography
