# Supplementary material for: Different Trafficking Phenotypes of Niemann-Pick C1 Gene Mutations Correlate with Various Alterations in Lipid Storage, Membrane Composition and Miglustat Amenability
Source: Int J Mol Sci. 2020 Mar 19;21(6):2101. doi: 10.3390/ijms21062101 (PMC7139583; doi:10.3390/ijms21062101)
Supplement: Supplementary file 1 [file ijms-21-02101-s001.pdf]

Supplementary material

Different trafficking phenotypes of Niemann-Pick C1 gene mutations correlate with various alterations in lipid storage, membrane composition and miglustat amenability

Graham Brogden, Hadeel Shammas, Friederike Walters, Katia Maalouf, Anibh M. Das,
Hassan Y. Naim , Sandra Rizk

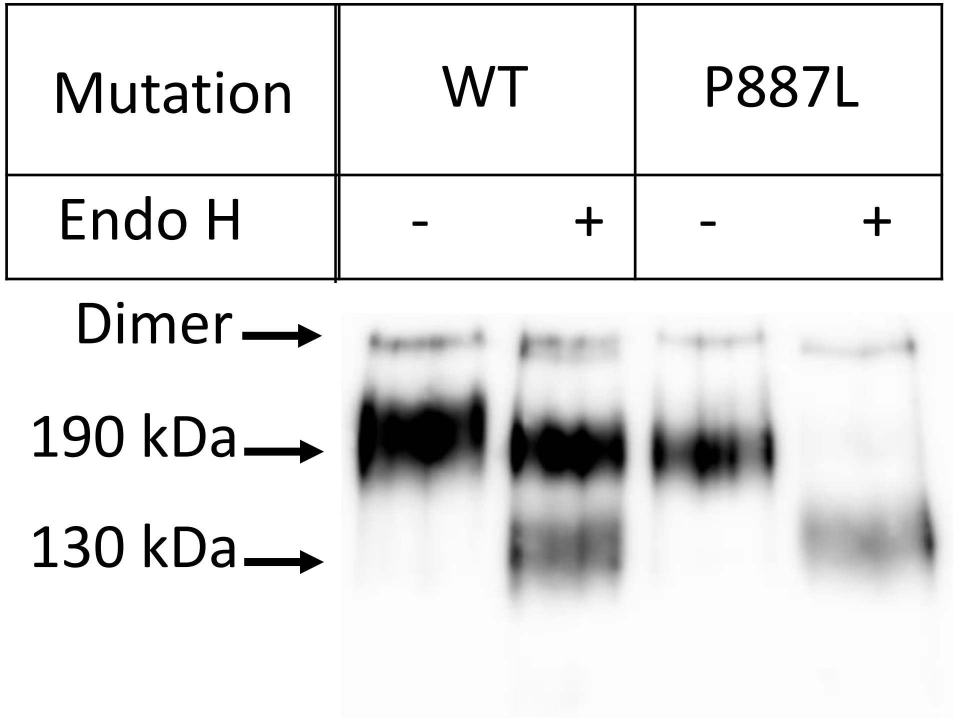

Supplementary 1

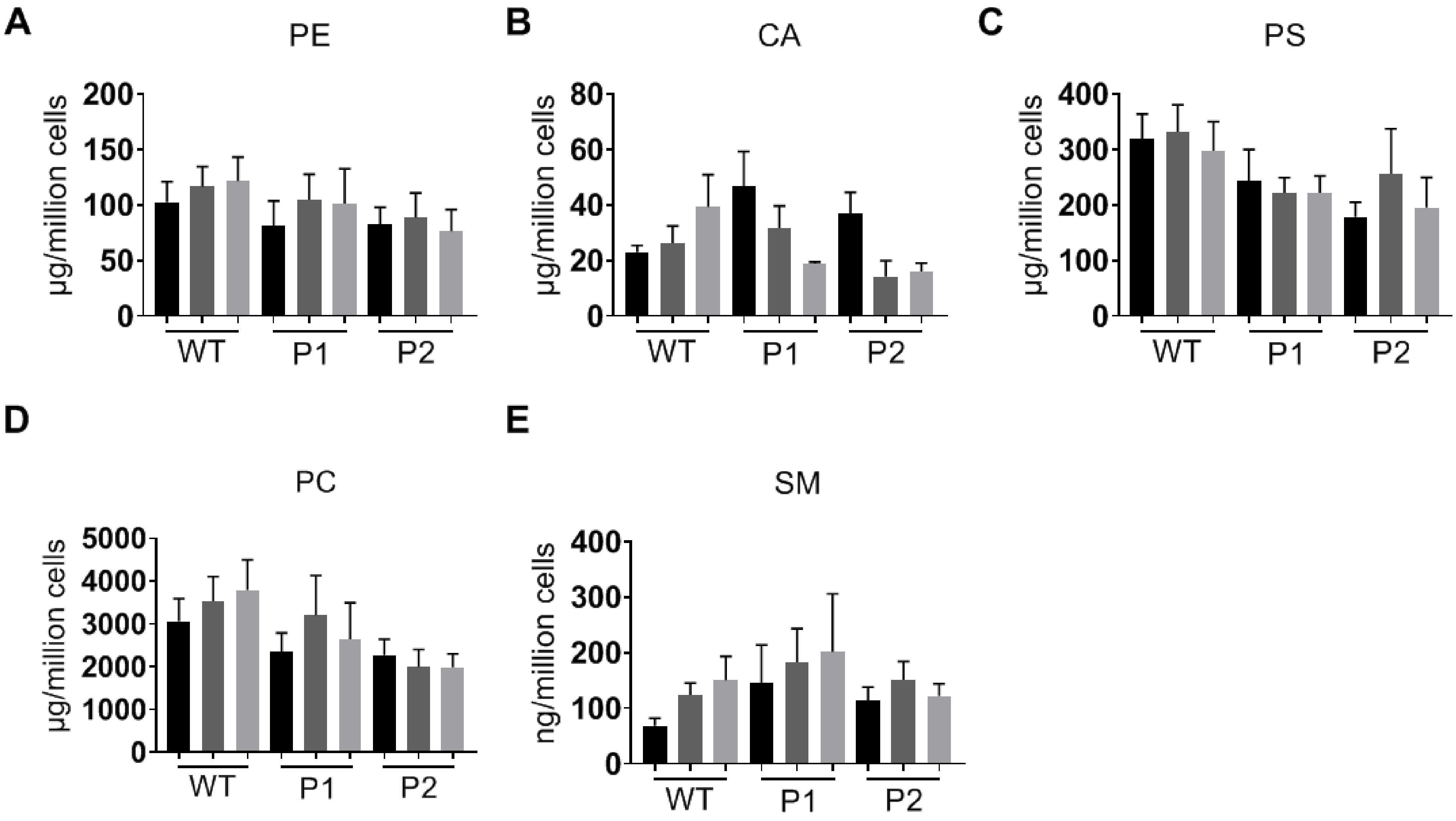

Supplementary 2

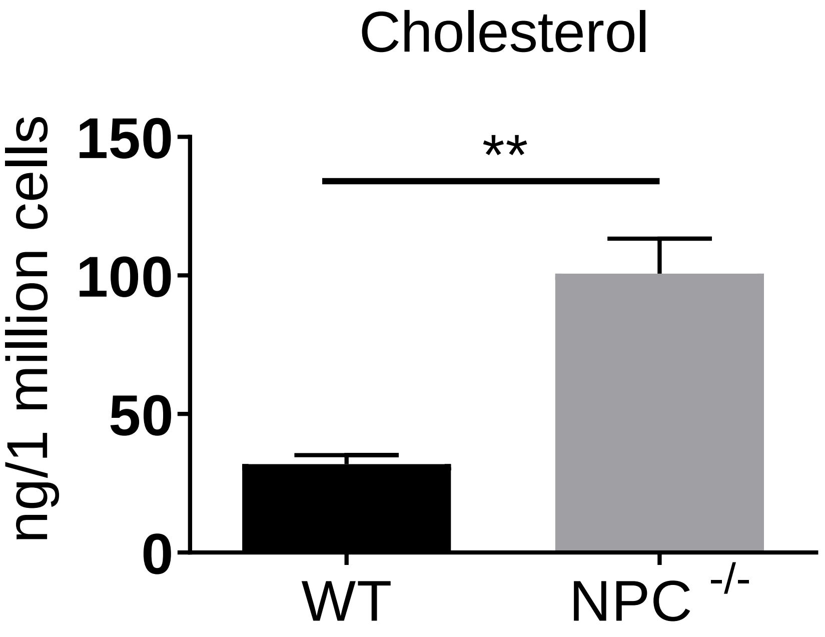

Supplementary 3

A

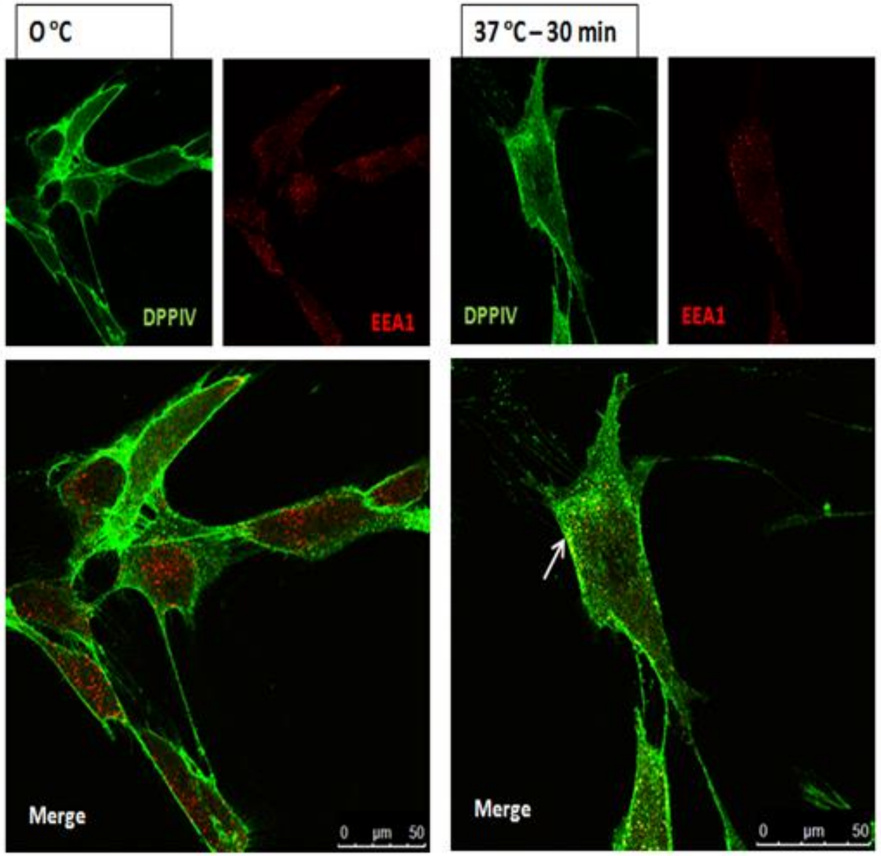

B

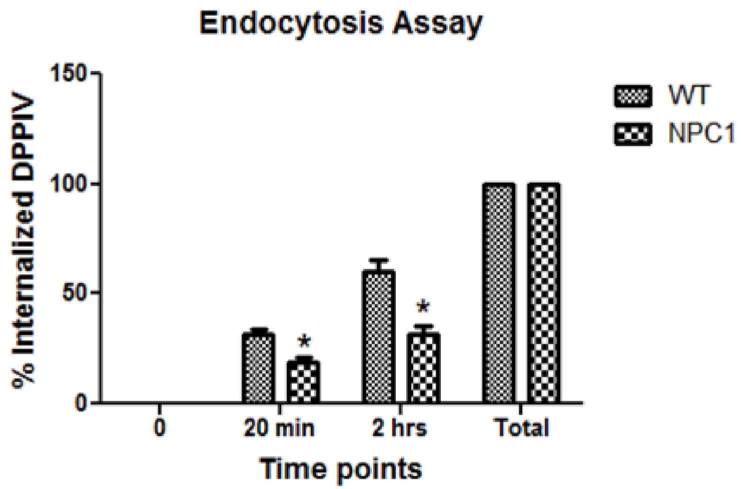

Supplementary 4

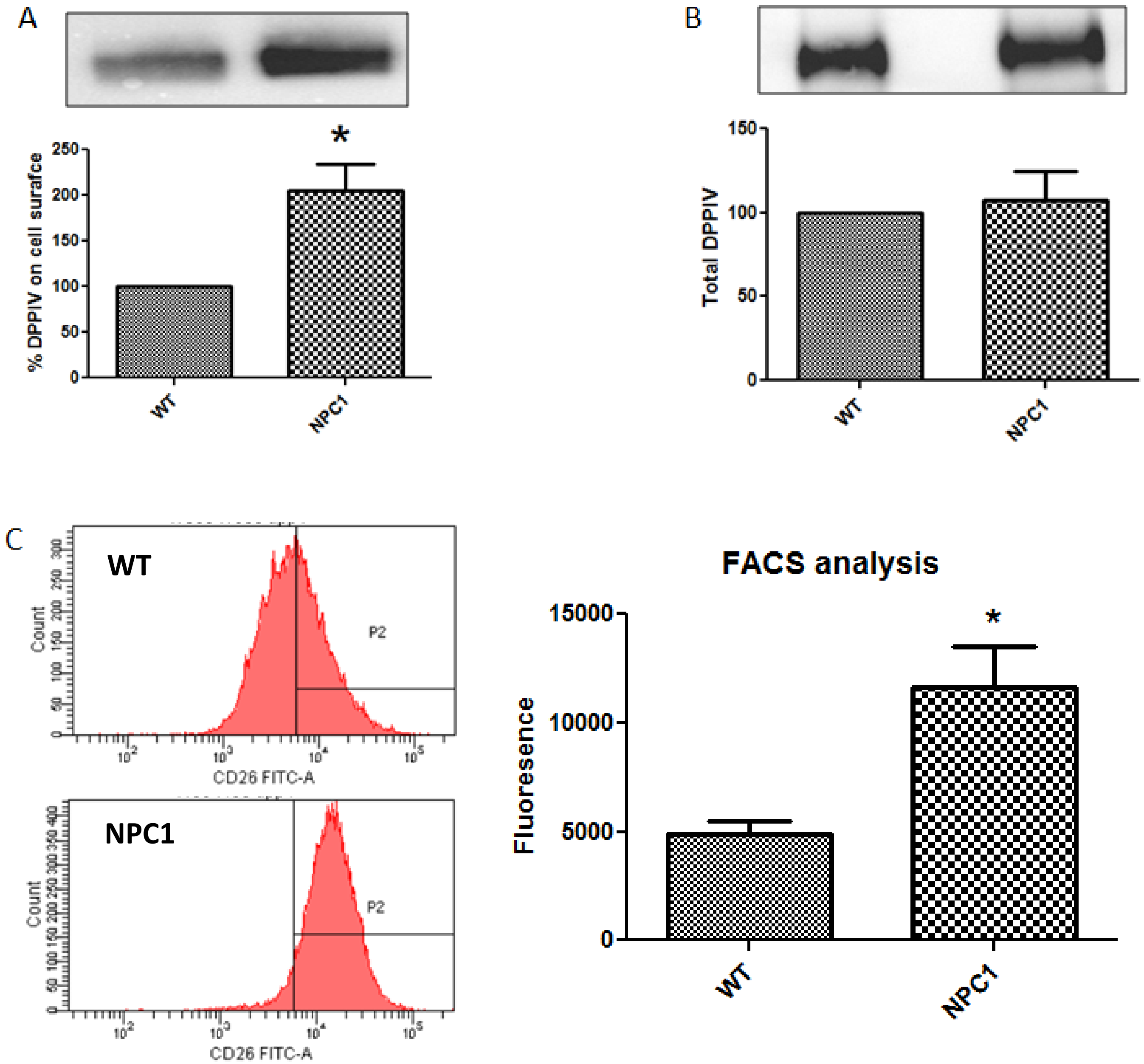

Supplementary 5

Legends of Supplementary Figures

Supplementary 1. COS-1 cells were transfected with a plasmid containing the NPC P887L construct. Homogenates were immunoprecipitated with an anti-NPC1 antibody, and the resulting sample was divided into two, one untreated (-) and the second treated with EndoH (+). The complex glycosylated EndoH-resistant form of the NPC protein can be seen at 190 kDa and the EndoH-sensitive form at 130 kDa.

Supplementary 2. Phospholipid composition of WT and patient fibroblasts determined by TLC. Each bar equates to at least 3 repetitions, with the WT corresponding to the average of 3 repetitions of 3 WT fibroblasts from independent healthy donors. P1: I1061T/P887L, P2: D874V/D948Y. Phosphoethanolamine (A), cardiolipin (B), phosphatidylserine (C), phosphatidylcholine (D) and sphingomyelin (E). SEM, student t-test.

Supplementary 3. CHO control and CHO $NPC^{-/-}$ cells were grown to confluence, washed, counted and lipids extracted. Cholesterol was measured by HPLC and the results expressed as ng cholesterol per $1x10^6$ cells. N=4, SEM, student t-test ** $P \leq 0.01$.

Supplementary 4. Delayed endocytosis of DPPIV in NPC cells. To determine the mode of DPPIV entry into fibroblasts, immunostaining was performed using an antibody against DPPIV (green) and EEA1 (red). Some cells were kept on ice whereas others were incubated at 37°C for 30 min. The co-localisation between the two proteins is depicted in the yellow vesicular structures appearing in the cells that were incubated at 37°C, indicating that DPPIV enters the cells through endocytosis. B) A biotin endocytosis assay was conducted for both WT and NPC cells. In this assay, biotin was used to label proteins on the surface at 4°C. Surface biotinylated cells were returned to a temperature of 37°C at different time points to allow the internalisation of the appropriate cell surface proteins with their biotin tag. Quantification of the endocytosis assay results revealed that after 2 h 28% of DPPIV was internalised in NPC cells compared to 56% in WT cells, indicating a two-fold delay in endocytosis of DPPIV in NPC cells. (* $p < 0.05$, n≥3 independent experiments).

Supplementary 5. DPPIV accumulates on cell surface of NPC cells. Cell surface protein analysis was performed by: A) biotin-avidin system based assay where isolated cell surface proteins were immunoblotted with a monoclonal DPPIV antibody, a representative immunoblot with only one of four WT cells is shown. No significant difference in the total DPPIV expression levels appeared when immunoblotting with the same DPPIV antibody was done against total WT and NPC cell lysates. (B) FACS analysis, where the increase in fluorescence signal emitted by NPC cells reflected an accumulation of DPPIV on the surface of NPC cells. (C) A 2-fold increase of this protein was observed on the membranes of NPC cells relative to the mean value ± SEM of four normal WT fibroblasts. (* $p < 0.05$, n≥3 independent experiments).
